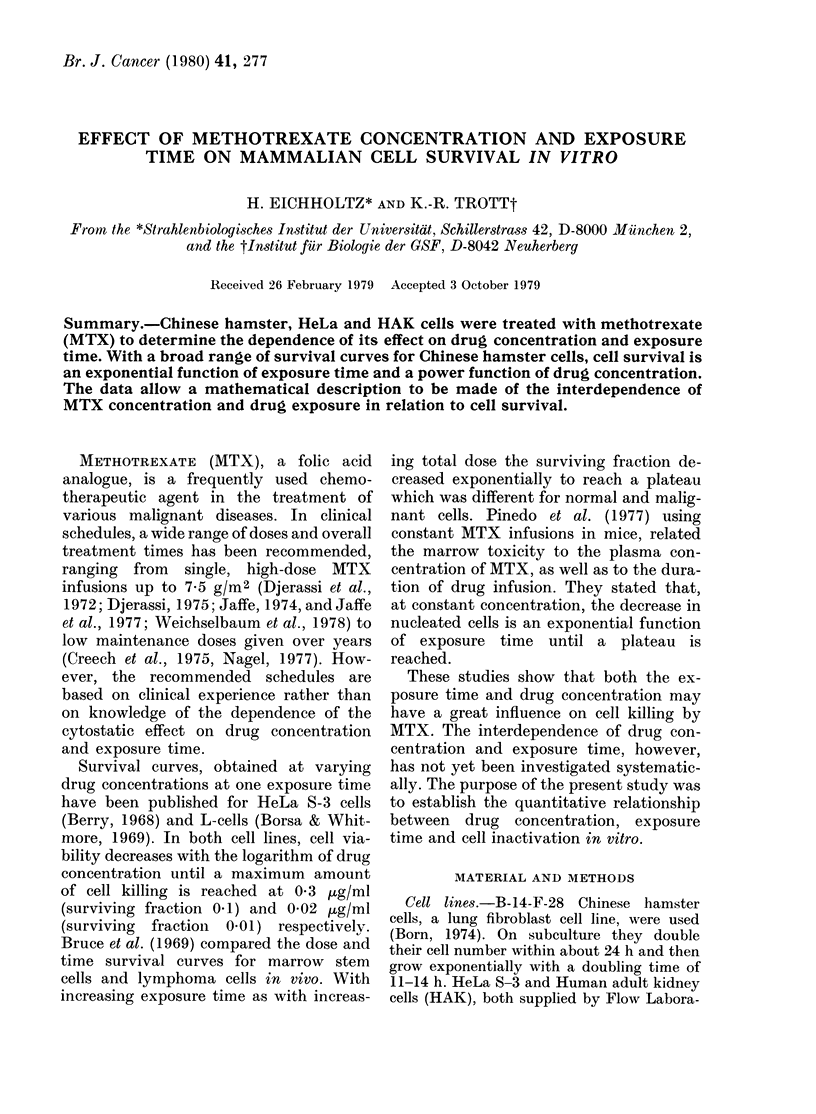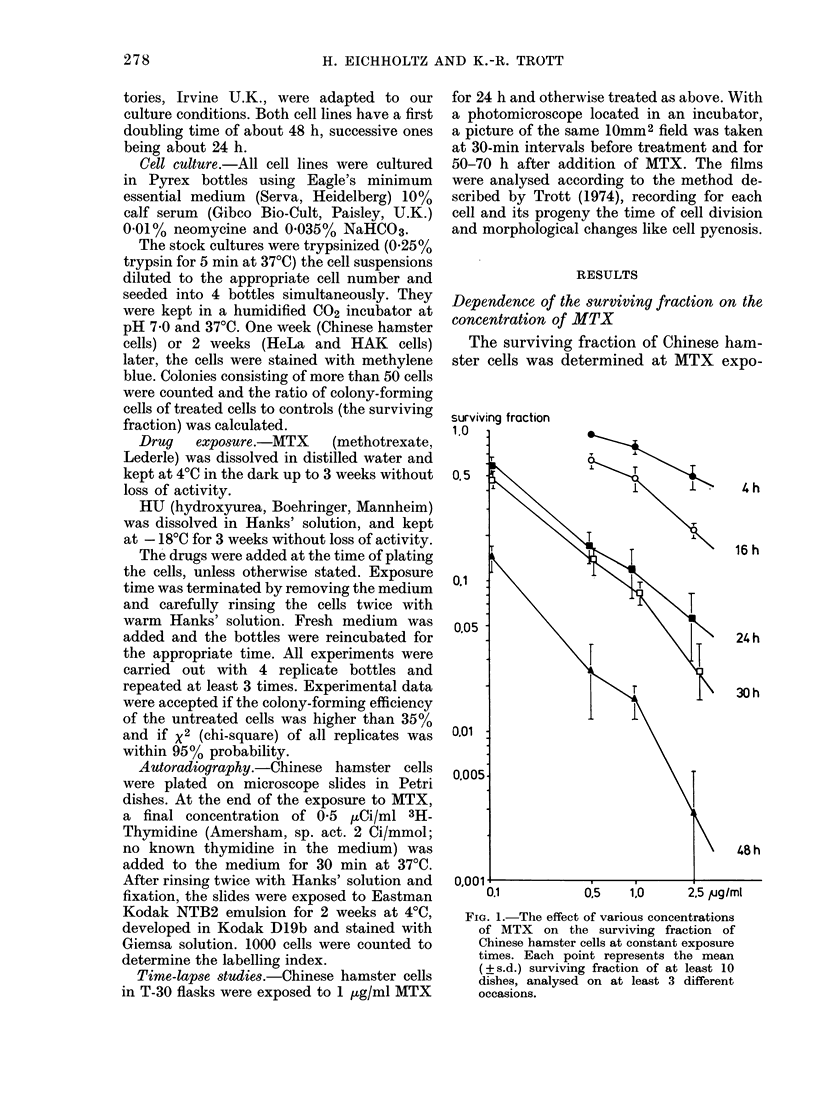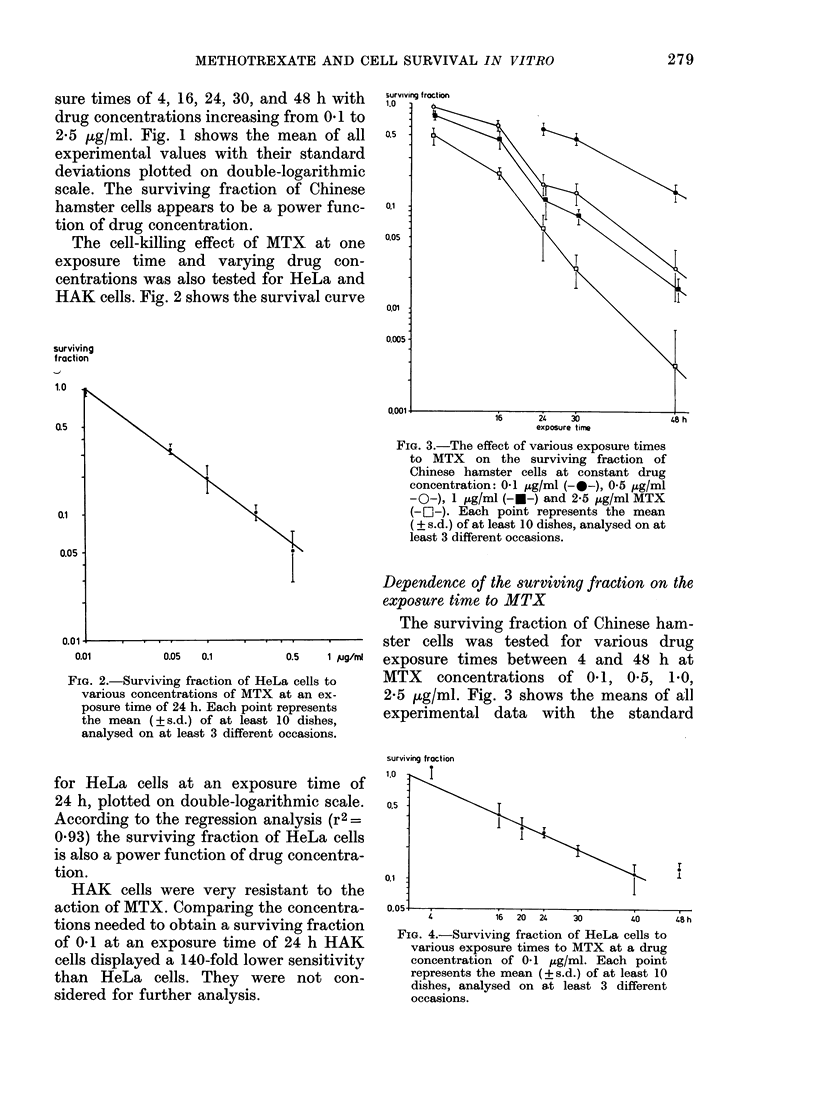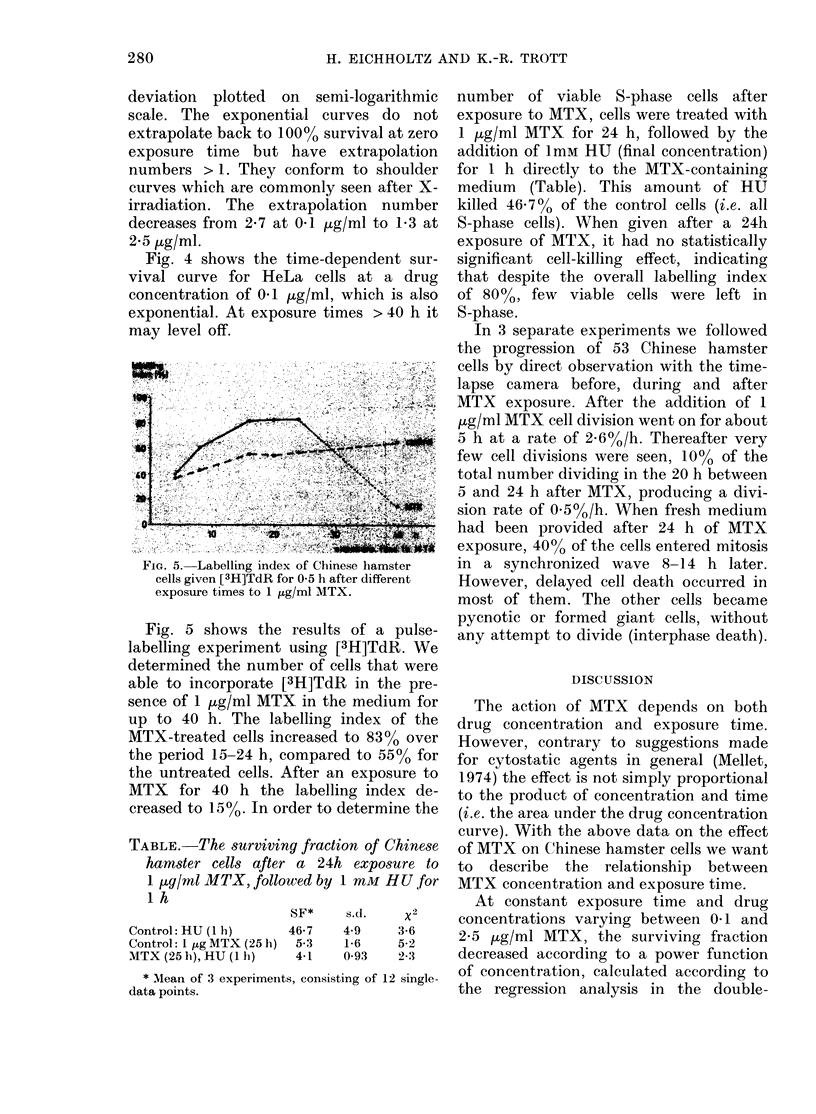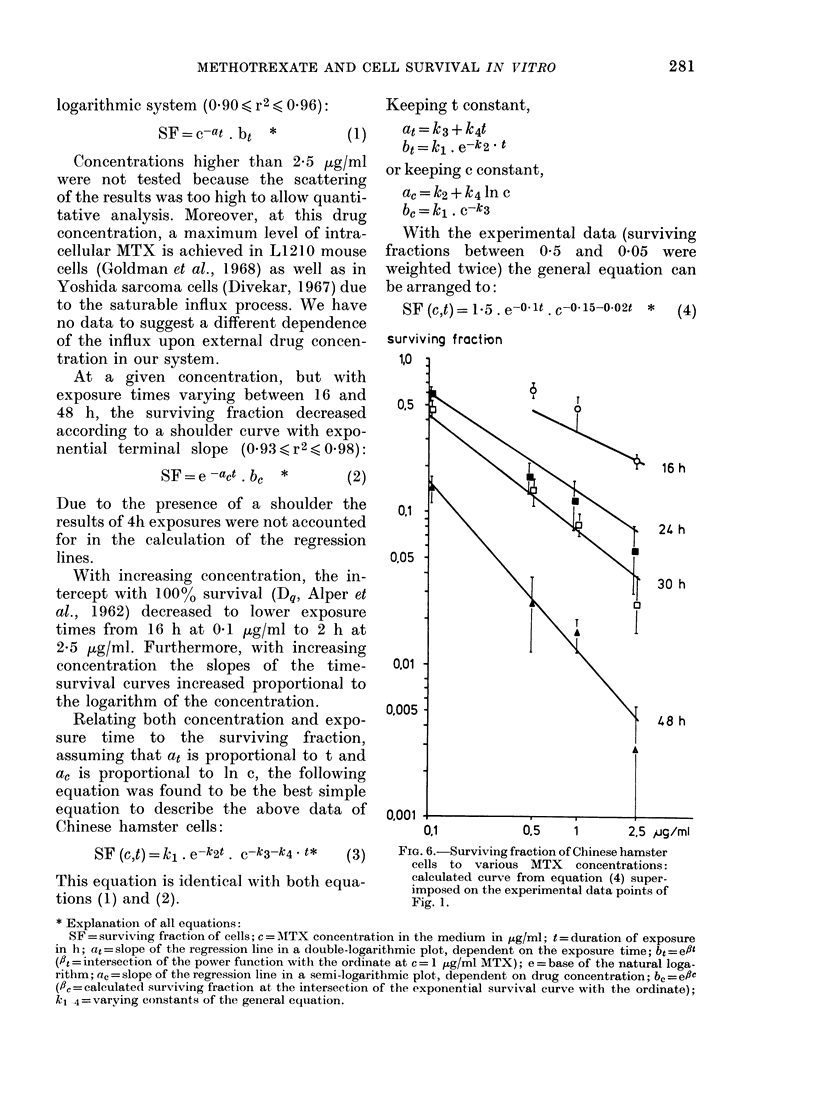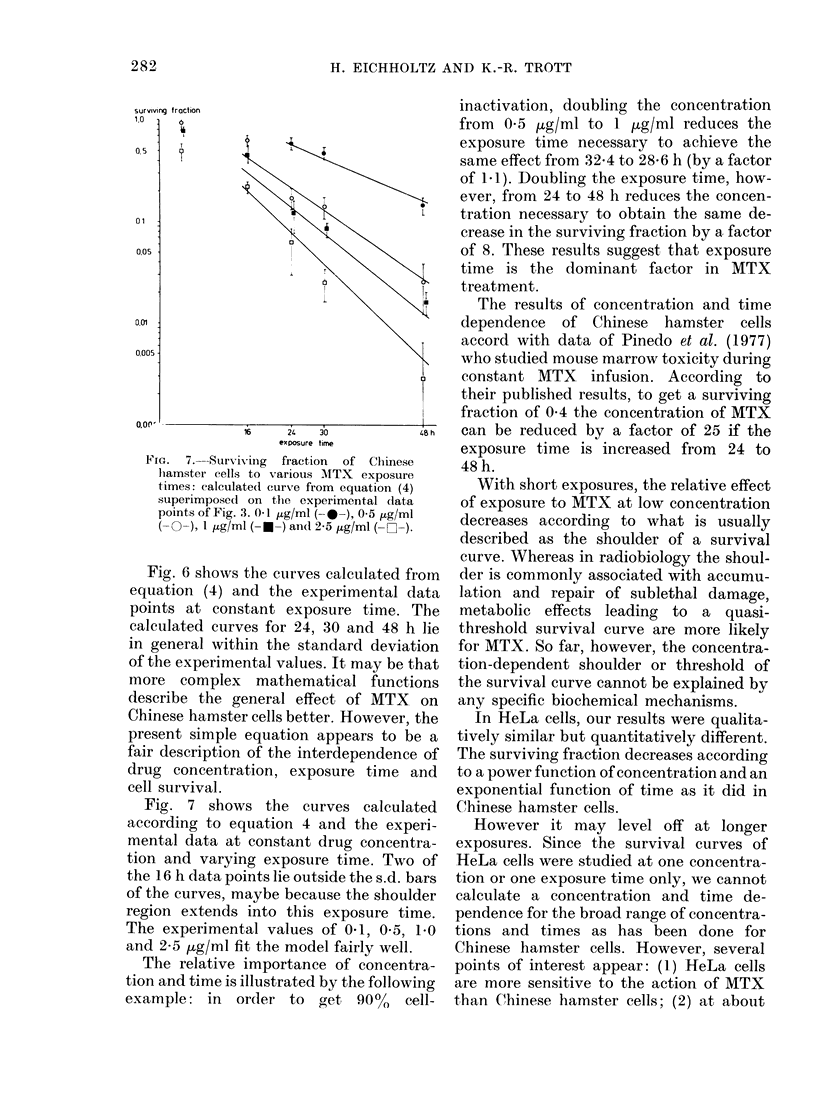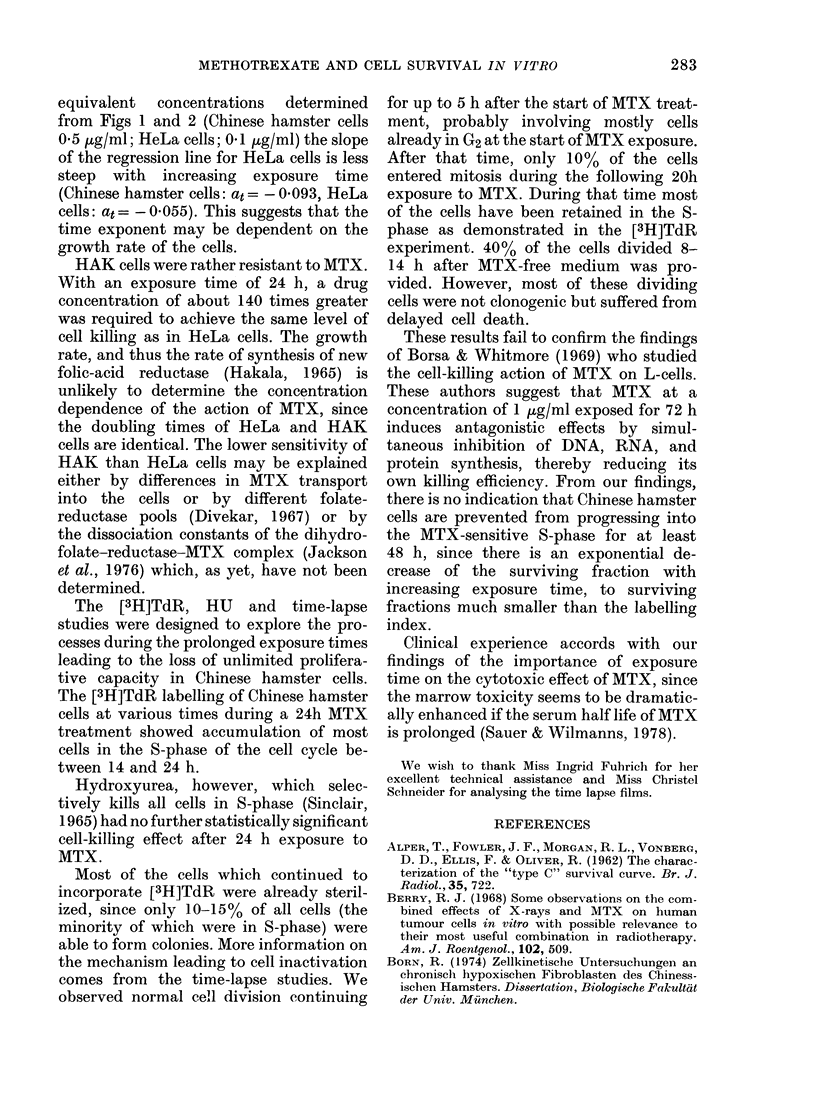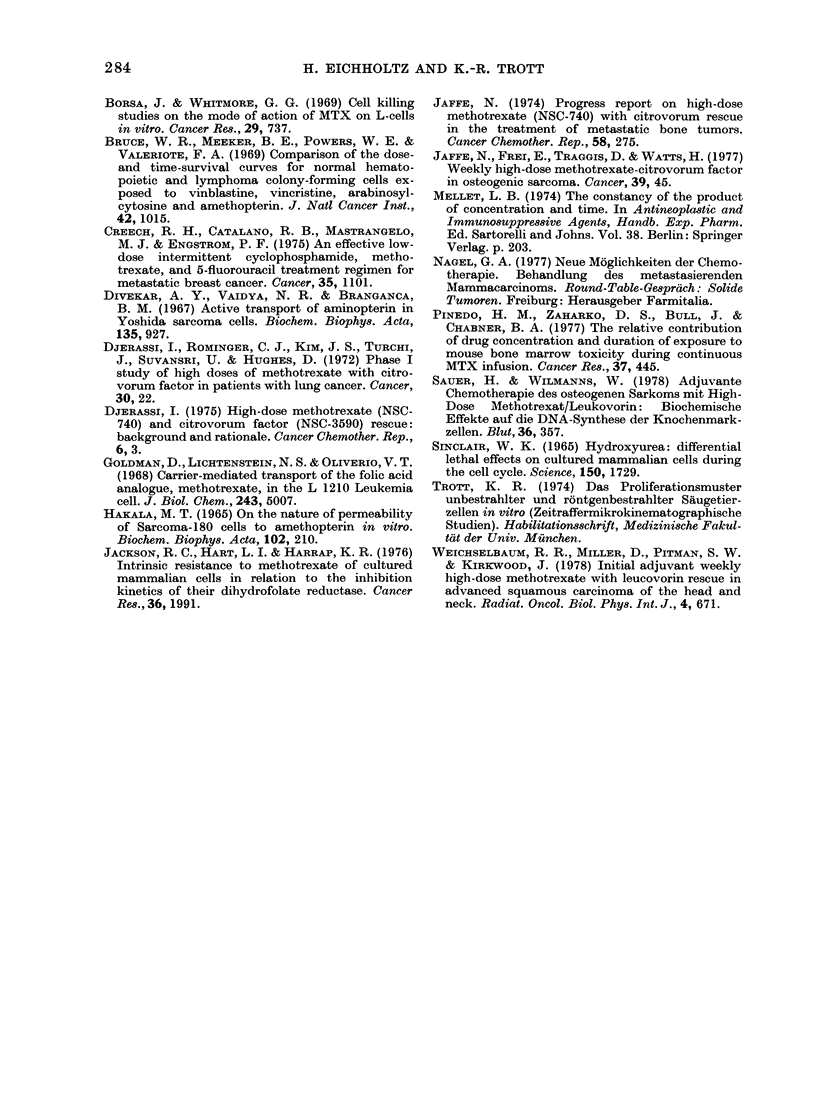# Effect of methotrexate concentration and exposure time on mammalian cell survival in vitro.

**DOI:** 10.1038/bjc.1980.40

**Published:** 1980-02

**Authors:** H. Eichholtz, K. R. Trott

## Abstract

Chinese hamster, HeLa and HAK cells were treated with methotrexate (MTX) to determine the dependence of its effect on drug concentration and exposure time. With a broad range of survival curves for Chinese hamster cells, cell survival is an exponential function of exposure time and a power function of drug concentration. The data allow a mathematical description to be made of the interdependence of MTX concentration and drug exposure in relation to cell survival.


*Br. J. Cancer* (1980) **41**, 277

### EFFECT OF METHOTREXATE CONCENTRATION AND EXPOSURE TIME ON MAMMALIAN CELL SURVIVAL *IN VITRO*

H. EICHHOLTZ* AND K.-R. TROTT†

*From the *Strahlenbiologisches Institut der Universität, Schillerstrass* 42, D-8000 *München* 2, *and the †Institut für Biologie der GSF*, *D*-8042 *Neuherberg*

Received 26 February 1979 Accepted 3 October 1979

**Summary.—Chinese hamster, HeLa and HAK cells were treated with methotrexate (MTX) to determine the dependence of its effect on drug concentration and exposure time. With a broad range of survival curves for Chinese hamster cells, cell survival is an exponential function of exposure time and a power function of drug concentration. The data allow a mathematical description to be made of the interdependence of MTX concentration and drug exposure in relation to cell survival.**

METHOTREXATE (MTX), a folic acid analogue, is a frequently used chemotherapeutic agent in the treatment of various malignant diseases. In clinical schedules, a wide range of doses and overall treatment times has been recommended, ranging from single, high-dose MTX infusions up to 7·5 g/m$^2$ (Djerassi *et al.*, 1972; Djerassi, 1975; Jaffe, 1974, and Jaffe *et al.*, 1977; Weichselbaum *et al.*, 1978) to low maintenance doses given over years (Creech *et al.*, 1975, Nagel, 1977). However, the recommended schedules are based on clinical experience rather than on knowledge of the dependence of the cytostatic effect on drug concentration and exposure time.

Survival curves, obtained at varying drug concentrations at one exposure time have been published for HeLa S-3 cells (Berry, 1968) and L-cells (Borsa & Whitmore, 1969). In both cell lines, cell viability decreases with the logarithm of drug concentration until a maximum amount of cell killing is reached at 0·3 μg/ml (surviving fraction 0·1) and 0·02 μg/ml (surviving fraction 0·01) respectively. Bruce *et al.* (1969) compared the dose and time survival curves for marrow stem cells and lymphoma cells *in vivo*. With increasing exposure time as with increasing total dose the surviving fraction decreased exponentially to reach a plateau which was different for normal and malignant cells. Pinedo *et al.* (1977) using constant MTX infusions in mice, related the marrow toxicity to the plasma concentration of MTX, as well as to the duration of drug infusion. They stated that, at constant concentration, the decrease in nucleated cells is an exponential function of exposure time until a plateau is reached.

These studies show that both the exposure time and drug concentration may have a great influence on cell killing by MTX. The interdependence of drug concentration and exposure time, however, has not yet been investigated systematically. The purpose of the present study was to establish the quantitative relationship between drug concentration, exposure time and cell inactivation *in vitro*.

### MATERIAL AND METHODS

*Cell lines.*—B-14-F-28 Chinese hamster cells, a lung fibroblast cell line, were used (Born, 1974). On subculture they double their cell number within about 24 h and then grow exponentially with a doubling time of 11–14 h. HeLa S–3 and Human adult kidney cells (HAK), both supplied by Flow Laboratories, Irvine U.K., were adapted to our culture conditions. Both cell lines have a first doubling time of about 48 h, successive ones being about 24 h.

*Cell culture.*—All cell lines were cultured in Pyrex bottles using Eagle's minimum essential medium (Serva, Heidelberg) 10% calf serum (Gibco Bio-Cult, Paisley, U.K.) 0·01% neomycine and 0·035% $NaHCO_3$.

The stock cultures were trypsinized (0·25% trypsin for 5 min at 37°C) the cell suspensions diluted to the appropriate cell number and seeded into 4 bottles simultaneously. They were kept in a humidified $CO_2$ incubator at pH 7·0 and 37°C. One week (Chinese hamster cells) or 2 weeks (HeLa and HAK cells) later, the cells were stained with methylene blue. Colonies consisting of more than 50 cells were counted and the ratio of colony-forming cells of treated cells to controls (the surviving fraction) was calculated.

*Drug exposure.*—MTX (methotrexate, Lederle) was dissolved in distilled water and kept at 4°C in the dark up to 3 weeks without loss of activity.

HU (hydroxyurea, Boehringer, Mannheim) was dissolved in Hanks' solution, and kept at −18°C for 3 weeks without loss of activity.

The drugs were added at the time of plating the cells, unless otherwise stated. Exposure time was terminated by removing the medium and carefully rinsing the cells twice with warm Hanks' solution. Fresh medium was added and the bottles were reincubated for the appropriate time. All experiments were carried out with 4 replicate bottles and repeated at least 3 times. Experimental data were accepted if the colony-forming efficiency of the untreated cells was higher than 35% and if $\chi^2$ (chi-square) of all replicates was within 95% probability.

*Autoradiography.*—Chinese hamster cells were plated on microscope slides in Petri dishes. At the end of the exposure to MTX, a final concentration of 0·5 μCi/ml $^3$H-Thymidine (Amersham, sp. act. 2 Ci/mmol; no known thymidine in the medium) was added to the medium for 30 min at 37°C. After rinsing twice with Hanks' solution and fixation, the slides were exposed to Eastman Kodak NTB2 emulsion for 2 weeks at 4°C, developed in Kodak D19b and stained with Giemsa solution. 1000 cells were counted to determine the labelling index.

*Time-lapse studies.*—Chinese hamster cells in T-30 flasks were exposed to 1 μg/ml MTX for 24 h and otherwise treated as above. With a photomicroscope located in an incubator, a picture of the same 10mm$^2$ field was taken at 30-min intervals before treatment and for 50–70 h after addition of MTX. The films were analysed according to the method described by Trott (1974), recording for each cell and its progeny the time of cell division and morphological changes like cell pycnosis.

### RESULTS

*Dependence of the surviving fraction on the concentration of MTX*

The surviving fraction of Chinese hamster cells was determined at MTX expo-

FIG. 1.—The effect of various concentrations of MTX on the surviving fraction of Chinese hamster cells at constant exposure times. Each point represents the mean (±s.d.) surviving fraction of at least 10 dishes, analysed on at least 3 different occasions.

sure times of 4, 16, 24, 30, and 48 h with drug concentrations increasing from 0·1 to 2·5 μg/ml. Fig. 1 shows the mean of all experimental values with their standard deviations plotted on double-logarithmic scale. The surviving fraction of Chinese hamster cells appears to be a power function of drug concentration.

The cell-killing effect of MTX at one exposure time and varying drug concentrations was also tested for HeLa and HAK cells. Fig. 2 shows the survival curve

FIG. 2.—Surviving fraction of HeLa cells to various concentrations of MTX at an exposure time of 24 h. Each point represents the mean (±s.d.) of at least 10 dishes, analysed on at least 3 different occasions.

for HeLa cells at an exposure time of 24 h, plotted on double-logarithmic scale. According to the regression analysis ($r^2 = 0{\cdot}93$) the surviving fraction of HeLa cells is also a power function of drug concentration.

HAK cells were very resistant to the action of MTX. Comparing the concentrations needed to obtain a surviving fraction of 0·1 at an exposure time of 24 h HAK cells displayed a 140-fold lower sensitivity than HeLa cells. They were not considered for further analysis.

FIG. 3.—The effect of various exposure times to MTX on the surviving fraction of Chinese hamster cells at constant drug concentration: 0·1 μg/ml (-●-), 0·5 μg/ml -○-), 1 μg/ml (-■-) and 2·5 μg/ml MTX (-□-). Each point represents the mean (±s.d.) of at least 10 dishes, analysed on at least 3 different occasions.

*Dependence of the surviving fraction on the exposure time to MTX*

The surviving fraction of Chinese hamster cells was tested for various drug exposure times between 4 and 48 h at MTX concentrations of 0·1, 0·5, 1·0, 2·5 μg/ml. Fig. 3 shows the means of all experimental data with the standard

FIG. 4.—Surviving fraction of HeLa cells to various exposure times to MTX at a drug concentration of 0·1 μg/ml. Each point represents the mean (±s.d.) of at least 10 dishes, analysed on at least 3 different occasions.

deviation plotted on semi-logarithmic scale. The exponential curves do not extrapolate back to 100% survival at zero exposure time but have extrapolation numbers > 1. They conform to shoulder curves which are commonly seen after X-irradiation. The extrapolation number decreases from 2·7 at 0·1 μg/ml to 1·3 at 2·5 μg/ml.

Fig. 4 shows the time-dependent survival curve for HeLa cells at a drug concentration of 0·1 μg/ml, which is also exponential. At exposure times > 40 h it may level off.

Fig. 5.—Labelling index of Chinese hamster cells given [$^3$H]TdR for 0·5 h after different exposure times to 1 μg/ml MTX.

Fig. 5 shows the results of a pulse-labelling experiment using [$^3$H]TdR. We determined the number of cells that were able to incorporate [$^3$H]TdR in the presence of 1 μg/ml MTX in the medium for up to 40 h. The labelling index of the MTX-treated cells increased to 83% over the period 15–24 h, compared to 55% for the untreated cells. After an exposure to MTX for 40 h the labelling index decreased to 15%. In order to determine the number of viable S-phase cells after exposure to MTX, cells were treated with 1 μg/ml MTX for 24 h, followed by the addition of 1mM HU (final concentration) for 1 h directly to the MTX-containing medium (Table). This amount of HU killed 46·7% of the control cells (*i.e.* all S-phase cells). When given after a 24h exposure of MTX, it had no statistically significant cell-killing effect, indicating that despite the overall labelling index of 80%, few viable cells were left in S-phase.

In 3 separate experiments we followed the progression of 53 Chinese hamster cells by direct observation with the time-lapse camera before, during and after MTX exposure. After the addition of 1 μg/ml MTX cell division went on for about 5 h at a rate of 2·6%/h. Thereafter very few cell divisions were seen, 10% of the total number dividing in the 20 h between 5 and 24 h after MTX, producing a division rate of 0·5%/h. When fresh medium had been provided after 24 h of MTX exposure, 40% of the cells entered mitosis in a synchronized wave 8–14 h later. However, delayed cell death occurred in most of them. The other cells became pycnotic or formed giant cells, without any attempt to divide (interphase death).

Table.—*The surviving fraction of Chinese hamster cells after a 24h exposure to 1 μg/ml MTX, followed by 1 mM HU for 1 h*

| | SF* | s.d. | $\chi^2$ |
|---|---|---|---|
| Control: HU (1 h) | 46·7 | 4·9 | 3·6 |
| Control: 1 μg MTX (25 h) | 5·3 | 1·6 | 5·2 |
| MTX (25 h), HU (1 h) | 4·1 | 0·93 | 2·3 |

* Mean of 3 experiments, consisting of 12 single-data points.

### DISCUSSION

The action of MTX depends on both drug concentration and exposure time. However, contrary to suggestions made for cytostatic agents in general (Mellet, 1974) the effect is not simply proportional to the product of concentration and time (*i.e.* the area under the drug concentration curve). With the above data on the effect of MTX on Chinese hamster cells we want to describe the relationship between MTX concentration and exposure time.

At constant exposure time and drug concentrations varying between 0·1 and 2·5 μg/ml MTX, the surviving fraction decreased according to a power function of concentration, calculated according to the regression analysis in the doublelogarithmic system ($0{\cdot}90 \leqslant r^2 \leqslant 0{\cdot}96$):

$$SF = c^{-a_t} \, . \, b_t \quad * \qquad (1)$$

Concentrations higher than 2·5 μg/ml were not tested because the scattering of the results was too high to allow quantitative analysis. Moreover, at this drug concentration, a maximum level of intracellular MTX is achieved in L1210 mouse cells (Goldman *et al.*, 1968) as well as in Yoshida sarcoma cells (Divekar, 1967) due to the saturable influx process. We have no data to suggest a different dependence of the influx upon external drug concentration in our system.

At a given concentration, but with exposure times varying between 16 and 48 h, the surviving fraction decreased according to a shoulder curve with exponential terminal slope ($0{\cdot}93 \leqslant r^2 \leqslant 0{\cdot}98$):

$$SF = e^{-a_c t} \, . \, b_c \quad * \qquad (2)$$

Due to the presence of a shoulder the results of 4h exposures were not accounted for in the calculation of the regression lines.

With increasing concentration, the intercept with 100% survival ($D_q$, Alper *et al.*, 1962) decreased to lower exposure times from 16 h at 0·1 μg/ml to 2 h at 2·5 μg/ml. Furthermore, with increasing concentration the slopes of the time-survival curves increased proportional to the logarithm of the concentration.

Relating both concentration and exposure time to the surviving fraction, assuming that $a_t$ is proportional to t and $a_c$ is proportional to ln c, the following equation was found to be the best simple equation to describe the above data of Chinese hamster cells:

$$SF\,(c,t) = k_1 \, . \, e^{-k_2 t} \, . \, c^{-k_3 - k_4 \cdot t} * \qquad (3)$$

This equation is identical with both equations (1) and (2).

Keeping t constant,

$$a_t = k_3 + k_4 t$$

$$b_t = k_1 \, . \, e^{-k_2 \cdot t}$$

or keeping c constant,

$$a_c = k_2 + k_4 \ln c$$
$$b_c = k_1 \, . \, c^{-k_3}$$

With the experimental data (surviving fractions between 0·5 and 0·05 were weighted twice) the general equation can be arranged to:

$$SF\,(c,t) = 1{\cdot}5 \, . \, e^{-0{\cdot}1t} \, . \, c^{-0{\cdot}15 - 0{\cdot}02t} \quad * \qquad (4)$$

FIG. 6.—Surviving fraction of Chinese hamster cells to various MTX concentrations: calculated curve from equation (4) superimposed on the experimental data points of Fig. 1.

* Explanation of all equations:

SF = surviving fraction of cells; $c$ = MTX concentration in the medium in μg/ml; $t$ = duration of exposure in h; $a_t$ = slope of the regression line in a double-logarithmic plot, dependent on the exposure time; $b_t = e^{\beta t}$ ($^{\beta}t$ = intersection of the power function with the ordinate at $c = 1$ μg/ml MTX); e = base of the natural logarithm; $a_c$ = slope of the regression line in a semi-logarithmic plot, dependent on drug concentration; $b_c = e^{\beta c}$ ($^{\beta}c$ = calculated surviving fraction at the intersection of the exponential survival curve with the ordinate); $k_{1-4}$ = varying constants of the general equation.

FIG. 7.—Surviving fraction of Chinese hamster cells to various MTX exposure times: calculated curve from equation (4) superimposed on the experimental data points of Fig. 3. 0·1 μg/ml (-●-), 0·5 μg/ml (-○-), 1 μg/ml (-■-) and 2·5 μg/ml (-□-).

Fig. 6 shows the curves calculated from equation (4) and the experimental data points at constant exposure time. The calculated curves for 24, 30 and 48 h lie in general within the standard deviation of the experimental values. It may be that more complex mathematical functions describe the general effect of MTX on Chinese hamster cells better. However, the present simple equation appears to be a fair description of the interdependence of drug concentration, exposure time and cell survival.

Fig. 7 shows the curves calculated according to equation 4 and the experimental data at constant drug concentration and varying exposure time. Two of the 16 h data points lie outside the s.d. bars of the curves, maybe because the shoulder region extends into this exposure time. The experimental values of 0·1, 0·5, 1·0 and 2·5 μg/ml fit the model fairly well.

The relative importance of concentration and time is illustrated by the following example: in order to get 90% cell-inactivation, doubling the concentration from 0·5 μg/ml to 1 μg/ml reduces the exposure time necessary to achieve the same effect from 32·4 to 28·6 h (by a factor of 1·1). Doubling the exposure time, however, from 24 to 48 h reduces the concentration necessary to obtain the same decrease in the surviving fraction by a factor of 8. These results suggest that exposure time is the dominant factor in MTX treatment.

The results of concentration and time dependence of Chinese hamster cells accord with data of Pinedo *et al.* (1977) who studied mouse marrow toxicity during constant MTX infusion. According to their published results, to get a surviving fraction of 0·4 the concentration of MTX can be reduced by a factor of 25 if the exposure time is increased from 24 to 48 h.

With short exposures, the relative effect of exposure to MTX at low concentration decreases according to what is usually described as the shoulder of a survival curve. Whereas in radiobiology the shoulder is commonly associated with accumulation and repair of sublethal damage, metabolic effects leading to a quasi-threshold survival curve are more likely for MTX. So far, however, the concentration-dependent shoulder or threshold of the survival curve cannot be explained by any specific biochemical mechanisms.

In HeLa cells, our results were qualitatively similar but quantitatively different. The surviving fraction decreases according to a power function of concentration and an exponential function of time as it did in Chinese hamster cells.

However it may level off at longer exposures. Since the survival curves of HeLa cells were studied at one concentration or one exposure time only, we cannot calculate a concentration and time dependence for the broad range of concentrations and times as has been done for Chinese hamster cells. However, several points of interest appear: (1) HeLa cells are more sensitive to the action of MTX than Chinese hamster cells; (2) at about equivalent concentrations determined from Figs 1 and 2 (Chinese hamster cells 0·5 μg/ml; HeLa cells; 0·1 μg/ml) the slope of the regression line for HeLa cells is less steep with increasing exposure time (Chinese hamster cells: $a_t = -0{\cdot}093$, HeLa cells: $a_t = -0{\cdot}055$). This suggests that the time exponent may be dependent on the growth rate of the cells.

HAK cells were rather resistant to MTX. With an exposure time of 24 h, a drug concentration of about 140 times greater was required to achieve the same level of cell killing as in HeLa cells. The growth rate, and thus the rate of synthesis of new folic-acid reductase (Hakala, 1965) is unlikely to determine the concentration dependence of the action of MTX, since the doubling times of HeLa and HAK cells are identical. The lower sensitivity of HAK than HeLa cells may be explained either by differences in MTX transport into the cells or by different folate-reductase pools (Divekar, 1967) or by the dissociation constants of the dihydrofolate–reductase–MTX complex (Jackson *et al.*, 1976) which, as yet, have not been determined.

The [$^3$H]TdR, HU and time-lapse studies were designed to explore the processes during the prolonged exposure times leading to the loss of unlimited proliferative capacity in Chinese hamster cells. The [$^3$H]TdR labelling of Chinese hamster cells at various times during a 24h MTX treatment showed accumulation of most cells in the S-phase of the cell cycle between 14 and 24 h.

Hydroxyurea, however, which selectively kills all cells in S-phase (Sinclair, 1965) had no further statistically significant cell-killing effect after 24 h exposure to MTX.

Most of the cells which continued to incorporate [$^3$H]TdR were already sterilized, since only 10–15% of all cells (the minority of which were in S-phase) were able to form colonies. More information on the mechanism leading to cell inactivation comes from the time-lapse studies. We observed normal cell division continuing for up to 5 h after the start of MTX treatment, probably involving mostly cells already in $G_2$ at the start of MTX exposure. After that time, only 10% of the cells entered mitosis during the following 20h exposure to MTX. During that time most of the cells have been retained in the S-phase as demonstrated in the [$^3$H]TdR experiment. 40% of the cells divided 8–14 h after MTX-free medium was provided. However, most of these dividing cells were not clonogenic but suffered from delayed cell death.

These results fail to confirm the findings of Borsa & Whitmore (1969) who studied the cell-killing action of MTX on L-cells. These authors suggest that MTX at a concentration of 1 μg/ml exposed for 72 h induces antagonistic effects by simultaneous inhibition of DNA, RNA, and protein synthesis, thereby reducing its own killing efficiency. From our findings, there is no indication that Chinese hamster cells are prevented from progressing into the MTX-sensitive S-phase for at least 48 h, since there is an exponential decrease of the surviving fraction with increasing exposure time, to surviving fractions much smaller than the labelling index.

Clinical experience accords with our findings of the importance of exposure time on the cytotoxic effect of MTX, since the marrow toxicity seems to be dramatically enhanced if the serum half life of MTX is prolonged (Sauer & Wilmanns, 1978).

We wish to thank Miss Ingrid Fuhrich for her excellent technical assistance and Miss Christel Schneider for analysing the time lapse films.

### REFERENCES

ALPER, T., FOWLER, J. F., MORGAN, R. L., VONBERG, D. D., ELLIS, F. & OLIVER, R. (1962) The characterization of the "type C" survival curve. *Br. J. Radiol.*, **35**, 722.

BERRY, R. J. (1968) Some observations on the combined effects of X-rays and MTX on human tumour cells *in vitro* with possible relevance to their most useful combination in radiotherapy. *Am. J. Roentgenol.*, **102**, 509.

BORN, R. (1974) Zellkinetische Untersuchungen an chronisch hypoxischen Fibroblasten des Chinessischen Hamsters. *Dissertation, Biologische Fakultät der Univ. München.*

BORSA, J. & WHITMORE, G. G. (1969) Cell killing studies on the mode of action of MTX on L-cells *in vitro*. *Cancer Res.*, **29**, 737.

BRUCE, W. R., MEEKER, B. E., POWERS, W. E. & VALERIOTE, F. A. (1969) Comparison of the dose- and time-survival curves for normal hematopoietic and lymphoma colony-forming cells exposed to vinblastine, vincristine, arabinosylcytosine and amethopterin. *J. Natl Cancer Inst.*, **42**, 1015.

CREECH, R. H., CATALANO, R. B., MASTRANGELO, M. J. & ENGSTROM, P. F. (1975) An effective low-dose intermittent cyclophosphamide, methotrexate, and 5-fluorouracil treatment regimen for metastatic breast cancer. *Cancer*, **35**, 1101.

DIVEKAR, A. Y., VAIDYA, N. R. & BRANGANCA, B. M. (1967) Active transport of aminopterin in Yoshida sarcoma cells. *Biochem. Biophys. Acta*, **135**, 927.

DJERASSI, I., ROMINGER, C. J., KIM, J. S., TURCHI, J., SUVANSRI, U. & HUGHES, D. (1972) Phase I study of high doses of methotrexate with citrovorum factor in patients with lung cancer. *Cancer*, **30**, 22.

DJERASSI, I. (1975) High-dose methotrexate (NSC-740) and citrovorum factor (NSC-3590) rescue: background and rationale. *Cancer Chemother. Rep.*, **6**, 3.

GOLDMAN, D., LICHTENSTEIN, N. S. & OLIVERIO, V. T. (1968) Carrier-mediated transport of the folic acid analogue, methotrexate, in the L 1210 Leukemia cell. *J. Biol. Chem.*, **243**, 5007.

HAKALA, M. T. (1965) On the nature of permeability of Sarcoma-180 cells to amethopterin *in vitro*. *Biochem. Biophys. Acta*, **102**, 210.

JACKSON, R. C., HART, L. I. & HARRAP, K. R. (1976) Intrinsic resistance to methotrexate of cultured mammalian cells in relation to the inhibition kinetics of their dihydrofolate reductase. *Cancer Res.*, **36**, 1991.

JAFFE, N. (1974) Progress report on high-dose methotrexate (NSC-740) with citrovorum rescue in the treatment of metastatic bone tumors. *Cancer Chemother. Rep.*, **58**, 275.

JAFFE, N., FREI, E., TRAGGIS, D. & WATTS, H. (1977) Weekly high-dose methotrexate-citrovorum factor in osteogenic sarcoma. *Cancer*, **39**, 45.

MELLET, L. B. (1974) The constancy of the product of concentration and time. In *Antineoplastic and Immunosuppressive Agents, Handb. Exp. Pharm.* Ed. Sartorelli and Johns. Vol. 38. Berlin: Springer Verlag. p. 203.

NAGEL, G. A. (1977) Neue Möglichkeiten der Chemotherapie. Behandlung des metastasierenden Mammacarcinoms. *Round-Table-Gespräch: Solide Tumoren.* Freiburg: Herausgeber Farmitalia.

PINEDO, H. M., ZAHARKO, D. S., BULL, J. & CHABNER, B. A. (1977) The relative contribution of drug concentration and duration of exposure to mouse bone marrow toxicity during continuous MTX infusion. *Cancer Res.*, **37**, 445.

SAUER, H. & WILMANNS, W. (1978) Adjuvante Chemotherapie des osteogenen Sarkoms mit High-Dose Methotrexat/Leukovorin: Biochemische Effekte auf die DNA-Synthese der Knochenmarkzellen. *Blut*, **36**, 357.

SINCLAIR, W. K. (1965) Hydroxyurea: differential lethal effects on cultured mammalian cells during the cell cycle. *Science*, **150**, 1729.

TROTT, K. R. (1974) Das Proliferationsmuster unbestrahlter und röntgenbestrahlter Säugetierzellen *in vitro* (Zeitraffermikrokinematographische Studien). *Habilitationsschrift, Medizinische Fakultät der Univ. München.*

WEICHSELBAUM, R. R., MILLER, D., PITMAN, S. W. & KIRKWOOD, J. (1978) Initial adjuvant weekly high-dose methotrexate with leucovorin rescue in advanced squamous carcinoma of the head and neck. *Radiat. Oncol. Biol. Phys. Int. J.*, **4**, 671.